# Therapeutic Strategies for Sleep Apnea in Hypertension and Heart Failure

**DOI:** 10.1155/2013/814169

**Published:** 2013-02-19

**Authors:** Akiko Noda, Seiko Miyata, Yoshinari Yasuda

**Affiliations:** ^1^Department of Biomedical Sciences, Chubu University, 1200 Matsumoto-cho, Kasugai-shi, Aichi 487-8501, Japan; ^2^Nagoya University Graduate School of Medicine, Nagoya 466-8560, Japan

## Abstract

Sleep-disordered breathing (SDB) causes hypoxemia, negative intrathoracic pressure, and frequent arousal, contributing to increased cardiovascular disease mortality and morbidity. Obstructive sleep apnea syndrome (OSAS) is linked to hypertension, ischemic heart disease, and cardiac arrhythmias. Successful continuous positive airway pressure (CPAP) treatment has a beneficial effect on hypertension and improves the survival rate of patients with cardiovascular disease. Thus, long-term compliance with CPAP treatment may result in substantial blood pressure reduction in patients with resistant hypertension suffering from OSAS. Central sleep apnea and Cheyne-Stokes respiration occur in 30–50% of patients with heart failure (HF). Intermittent hypoxemia, nocturnal surges in sympathetic activity, and increased left ventricular preload and afterload due to negative intrathoracic pressure all lead to impaired cardiac function and poor life prognosis. SDB-related HF has been considered the potential therapeutic target. CPAP, nocturnal O_2_ therapy, and adaptive servoventilation minimize the effects of sleep apnea, thereby improving cardiac function, prognosis, and quality of life. Early diagnosis and treatment of SDB will yield better therapeutic outcomes for hypertension and HF.

## 1. Introduction

Obstructive sleep apnea syndrome (OSAS) is characterized by recurrent episodes of sleep apnea accompanied by hypoxia, fluctuations in heart rate and blood pressure (BP), frequent arousal, and consequent sleep fragmentation, resulting in an activation of the sympathetic nervous system [1–7]. The most common neuropsychiatric manifestation of OSAS is excessive daytime sleepiness that is secondary to sleep fragmentation and the lack of slow-wave sleep; other major, long-term manifestations of OSAS include disorders of the cardiovascular system [2].

The Sleep Heart Health Study [8] and Wisconsin Sleep Cohort Study [9] reported that OSAS is an independent risk factor for the development of essential hypertension. According to the Seventh Report of the Joint National Committee on Prevention, Detection, Evaluation, and Treatment of High Blood Pressure, OSAS is one of the identifiable causes of hypertension [10].

Central sleep apnea (CSA) and Cheyne-Stokes respiration (CSR) occur in 30–50% of patients with left ventricular (LV) dysfunction and heart failure (HF) caused by hypertension, cardiomyopathy, and ischemic heart disease [11]. Treatment of sleep apnea with nocturnal continuous positive airway pressure (CPAP) in individuals with congestive HF not only treats sleep-disordered breathing (SDB), but also results in improved LV function, alleviated HF symptoms, and reduced sympathetic activation due to decreased norepinephrine secretion [12]. Although CPAP reportedly improves LV function and survival rate in patients with HF [12, 13], outcomes of long-term compliance with CPAP treatment in these patients remain unknown. Moreover, guidelines for CPAP treatment have yet to be established.

## 2. Hypertension

OSAS and hypertension are often comorbid; approximately 50% of patients with OSAS are hypertensive, whereas 30–40% of patients with hypertension are estimated to suffer from OSAS [14]. The Sleep Heart Health Study in 2000, a cross-sectional study, reported that the prevalence of hypertension was independently associated with apnea-hypopnea index (AHI), after adjusting for obesity, alcohol intake, smoking habit, neck circumference, and waist-to-hip ratio [8]. The incidence of hypertension during a 4-year follow-up of the Wisconsin Sleep Cohort Study [9] rose with increasing OSAS severity. 

Patients with severe OSAS exhibit attenuated nocturnal BP dipping (nondipping pattern), as well as marked and rapid BP elevation in the morning shortly after waking (morning BP surge) [4, 6, 15]. Mounting evidence suggests that morning BP surge is significantly associated with cardiac, cerebral, renal, and vascular damage [16]. Cross-sectional studies have demonstrated consistent results that moderate-to-severe OSAS (AHI >15 events/h) increases the risk of developing hypertension [17]. Thus, the diagnosis of OSAS and the proper treatment of BP are of particular importance for patients with hypertension. 

We previously reported that repeated episodes of end-apneic arousal or hypoxia and consequent sleep fragmentation were associated with an increase in nocturnal BP, possibly leading to sustained hypertension and LV hypertrophy [4–6]. Both hypertension and LV hypertrophy are strong cardiovascular risk factors [18] and are linked to poor prognoses in patients with OSAS [19]. Yet, the mechanisms of how recurrent nocturnal BP surges and sympathetic activity lead to cardiovascular disease (CVD) or abnormal autonomic control of heart rate during the daytime in OSAS patients remains unclear. We also reported that nocturnal oxygen desaturation, sleep fragmentation, and increased sympathetic activity impaired daytime baroreflex sensitivity and nitric oxide (NO) production in patients with moderate-to-severe OSAS [20, 21]. Alterations in cardiac autonomic nervous function and endothelial dysfunction likely contribute to an increased risk of cardiovascular morbidity and mortality in OSAS patients.

Arousals that occur to terminate apneic events split the sleep pattern in patients with OSAS. Hypoxemia, CO_2_ retention, and respiratory acidosis caused by repeated apnea/hypopnea disrupt the normal cardiovascular system [3]. Hypoxemia, negative intrathoracic pressure, and increased sympathetic activity that accompany arousal are important factors in the development of hypertension in patients with OSAS [7]. Negative intrathoracic pressure during apnea increases LV afterload and elevates myocardial oxygen demand. OSAS also reduces parasympathetic activity and the baroreceptor reflex. Moreover, repetitive hypoxemia and reoxygenation induce oxidative stress. Endothelial dysfunction, augmentation of central aortic pressure and increased central pulse pressure, enhanced renin-angiotensin system (RAS) activity, and insulin resistance have been reported in patients with OSAS [20, 21]. The prevalence of metabolic syndrome in patients with OSAS is about twice that of those without OSAS [22]. Hypoxemia, sympathetic activation, chemoreceptor stimulation, and enhanced RAS in OSAS are possible mechanisms underlying the development of hypertension and target-organ damage.

CPAP treatment significantly reduces apnea/hypopnea, improves hypoxemia, decreases arousal, and normalizes sleep architecture. Severe OSAS significantly increases the risk of fatal and nonfatal cardiovascular events, and CPAP treatment reduces this risk [19]. These positive effects are observed from the first night of CPAP treatment in patients with OSAS; in a previous study, the 24-hour urinary excretion of norepinephrine was significantly reduced, and the plasma concentration of NO significantly increased, one night after CPAP treatment in patients with OSAS [21]. Successful CPAP treatment also ameliorates daytime excessive sleepiness, nocturnal arousal, and headache after waking. Furthermore, CPAP treatment reduces cyclic changes in heart beat and BP accompanied by apnea, increases arterial blood gas during waking, decreases brachial-ankle pulse wave velocity and the baroreceptor reflex, and decreases BP [5, 21]. Hence, CPAP not only treats SDB, but also improves the quality of life (QOL) by eliminating complications of OSAS and normalizing sleep architecture.

The effects of CPAP on BP reduction are observed both during daytime and sleep in patients with OSAS. Patients with resistant hypertension and OSAS showed a greater reduction in daytime diastolic BP, 24-hour systolic BP, and 24-hour diastolic BP after a 3-month CPAP treatment (>5.8 hours/night) [23]. Thus, 24-hour BP monitoring would be beneficial when starting CPAP treatment in patients undergoing antihypertensive therapy. Resistant hypertension increases cardiovascular risk, and thus it is an important public health problem. OSAS should be considered a major underlying factor that contributes to resistant hypertension [24–27]. 

OSAS is involved in CVD death; however, successful CPAP treatment reduces this risk [28, 29]. A cohort study in Japan reported that the prognosis in the middle-aged population might be affected by complications of hypertension or severity of oxygen desaturation related to OSAS [25]. The effects of CPAP treatment on systolic and diastolic BPs are related to daytime sleepiness and CPAP compliance [30, 31]. Appropriate treatment of OSAS should therefore be targeted to the prevention of hypertension, which causes CVD, as well as the early detection of atherosclerosis.

## 3. Heart Failure 

Patients with HF have a high prevalence of CSA/CSR, which is characterized by the oscillation of tidal volumes, resulting in PaCO_2_ levels below the apneic threshold. The overall mechanisms underlying CSA/CSR are complex, involving chemoreflexes by hypoxemia, prolonged circulation time by reduced cardiac output, upper airway resistance by congestion, and edema and arousals by hyperventilation and low PaCO_2_ [32–34]. Important risk factors of CSA/CSR include sex (male), age (>60 years old), PaCO_2_ (<38 mmHg), and a history of atrial fibrillation [35–39]. Poor survival rates among patients with HF are proportional to the frequency of central apneic events [40]. Along with CSA, low diastolic BP and severe right ventricular dysfunction are also variables predicting survival [41]. Given the high prevalence of SDB in men presenting with mild symptoms of HF, routine screening for SDB should be performed, as CPAP is a well-established treatment for such individuals.

CPAP treatment is effective in alleviating CSA/CSR in patients with HF [42–44]. Although the underlying mechanisms are unclear, the decrease in LV preload and afterload and increase in PaCO_2_ are possible contributing factors. CPAP treatment reduces cardiac output in patients with a pulmonary artery wedge pressure of <12 mmHg or normal cardiac output and stroke volume. In these patients, CPAP treatment should be carefully handled and followed up. In a randomized, controlled trial of 66 patients with CHF (29 with and 37 without CSR/CSA) under the age of 75 years, patients with CSR/CSA had a significantly increased mortality and cardiac transplantation rate compared with those without CSR/CSA [43]. Bradley et al. [12] found that CPAP resulted in attenuated CSA, improved nocturnal oxygenation, increased LV ejection fraction, lowered norepinephrine levels, and extended distance walked in six minutes; however, it did not affect patient survival. On the other hand, the Canadian CPAP for Patients with CSA and Heart Failure Trial (CANPAP) showed that CPAP might improve both LV ejection fraction and heart transplant-free survival if CSA is suppressed soon after its initiation [13]. Two studies have also assessed the role of CPAP in the prognosis of patients with OSAS and HF [45, 46].

 Bilevel positive airway pressure (BIPAP) can set the inspiratory and expiratory pressure levels separately, and thus there is less patient discomfort in BIPAP than with CPAP treatment. Moreover, BIPAP treatment improves lung compliance and ventilation-perfusion ratio. In our cohort study of 52 patients with idiopathic dilated cardiomyopathy and CSA, LV ejection fraction, deceleration time of the peak early LV filling velocity, and specific activity scale were significantly increased, and the LV end-systolic internal dimension, heart rate, systolic and diastolic BPs, and plasma BNP levels were all significantly decreased in 10 patients after a 3-month BIPAP treatment. There were no significant changes in any of the measured parameters in patients with AHI >20 events/h and drug treatment during the 3-month period. Moreover, four patients in this group died from worsening HF [47]. BIPAP treatment could be effective in patients with cardiac dysfunction/HF complicated with SDB [47–49] and should be considered a nonpharmacologic adjunct to conventional drug therapy.

Adaptive servoventilation (ASV) is a new approach to treating CSA/CSR and involves providing patients a small but varying amount of ventilatory support. One night of ASV suppresses CSA/CSR in patients with HF and improves sleep quality better than CPAP or 2 L/min of oxygen supplementation [50]. Given the lack of consensus on what constitutes the optimal therapy, further studies will be needed to establish the standard treatment strategy for CSA/CSR in patients with HF.

 Nocturnal oxygen supplementation reportedly diminishes CSR in patients with stable chronic HF [51]. Patients with HF, CSR, and hypoxemia related to CSA are considered candidates for nocturnal oxygen therapy; it is indicated for cases where CPAP treatment cannot achieve SpO_2_>90%, and oxygen increases until SpO_2_ reaches >90%. Nocturnal oxygen therapy prevents hypoxemia during apnea, lessens the direct adverse effects on the cardiovascular system, inhibits central chemoreceptor sensitivity to CO_2_, decreases sleep apnea by correcting hyperventilation during daytime, and inhibits sympathetic activity. Therefore, patient QOL ameliorates along with improvements in sleep architecture, shortness of breath and fatigue during daytime, and cognitive function [52]. Nocturnal oxygen therapy might also have long-term efficacy, as sympathetic activity is decreased in patients with chronic HF and CSA [53].

Angiotensin-converting enzyme inhibition can improve AHI and nocturnal oxygen desaturation in patients with mild-to-moderate HF [54]. Diuresis with a reduction in LV filling pressure has also been shown to lower the severity of CSA [55]. *β*-adrenergic blockade, which counters excess sympathetic activation and may modulate ventilatory responses in HF, was shown to decrease AHI in patients with CSA [56].

The evaluation of SDB in patients with CVD is critical in cardiovascular medicine. CSA/CSR may play an important role in HF progression, morbidity, and mortality. Although advances in medicine have increased the survival rates of patients with CVD, the recent increase in the number of patients with HF is a major concern. The development of therapeutic strategies for SDB-related HF will continue to be a challenging issue in clinical medicine.
